# Protein content and *HvNAM* alleles in Nordic barley (*Hordeum vulgare*) during a century of breeding

**DOI:** 10.1186/s41065-022-00227-y

**Published:** 2022-01-30

**Authors:** Jenny Hagenblad, Tytti Vanhala, Sharmila Madhavan, Matti W. Leino

**Affiliations:** 1 IFM Biology, Linköping University, SE-581 83 Linköping, Sweden; 2 Department of Animal Breeding and Genetics, Swedish University of Agricultural Sciences, SE-750 07 Uppsala, Sweden; 3 Mendel Centre for Plant Genomics and Proteomics, Central European Institute of Technology (CEITEC), Masaryk University, Kamenice 5, CZ- 625 00 Brno, Czech Republic; 4 The Archaeological Research Laboratory, Department of Archaeology and Classical Studies, Stockholm University, SE-106 91 Stockholm, Sweden

**Keywords:** *HvNAM*, Resequencing, Grain protein content, *Hordeum vulgare*, Crop improvement

## Abstract

**Background:**

Barley has been bred for more than a century in the Nordic countries, with dramatic improvements of yield traits. In this study we investigate if this has come at the cost of lower grain protein and micronutrient (iron, zinc) content, by analysing 80 accessions representing four different improvement stages. We further re-sequenced the two grain protein content associated genes *HvNAM-1* and *HvNAM-2* in full and performed expression analyses of the same genes to search for genetic associations with nutrient content.

**Results:**

We found higher thousand grain weight in barley landraces and in accessions from the late improvement group compared to accessions from the mid of the twentieth century. Straw length was much reduced in late stage accessions. No significant temporal decrease in grain protein, iron or zinc content during twentieth century Nordic crop improvement could be detected. Out of the 80 accessions only two deviant *HvNAM-1* sequences were found, represented by one accession each. These do not appear to be correlated to grain protein content. The sequence of *HvNAM-2* was invariable in all accessions and no correlations between expression levels of *HvNAM-1* and *HvNAM-2* and with grain protein content was found.

**Conclusions:**

In contrast to studies in wheat, where a strong negative correlation between straw length and grain protein and micronutrient content has been found, we do not see this relationship in Nordic barley. The last 60 years of breeding has reduced straw length but, contrary to expectations, not protein and micronutrient content. Variation in grain protein and micronutrient content was found among the Nordic barley accessions, but it is not explained by variation of *HvNAM* genes. This means that *HvNAM* is an unexploited source of genetic variation for nutrient content in Nordic barley.

**Supplementary Information:**

The online version contains supplementary material available at 10.1186/s41065-022-00227-y.

## Introduction

Barley, *Hordeum vulgare*, is a major cereal crop with a world-wide production of 141 million tonnes in 2018 (FAOSTAT, http://www.fao.org/faostat/en/). Although some barley grain is used for human consumption its main uses are as animal feed and for malting, with the use for malting and brewing being the most economically important [1]. Barley intended for different uses require different levels of protein in the grain, and, although other protein sources normally complement barley when used as the main carbohydrate source, a high protein content would result in high food and feed quality. In contrast, in malting and brewing too high protein content results in a slow and uneven water uptake during the germination process which lowers the amount of malt extract [2]. In addition, a high protein content in malting barley results in cloudiness in the beer and a reduced shelf life, although sufficient grain protein is needed for the yeast to grow during fermentation and for the production of preferred foam qualities. A suitably low or moderate protein content is thus required from quality malting barley [3].

Grain protein content (GPC) in barley is influenced both by environmental and genetic factors [4, 5]. Mapping studies of GPC points to polygenic control of the trait [3, 6]. In particular, two homologs of the well-studied wheat *NAM-B1*, *HvNAM-1* and *-2,* have been shown to be associated with GPC [6–10]. In wheat, the *NAM-B1* (*Gpc-B1*) gene has been shown to influence GPC. The gene codes for a NAC transcription factor which has been shown to have pleiotropic effects [11]. The wild-type allele facilitates the remobilisation of nutrients from flag leaves into the maturing grain and increases protein and mineral content while null mutations delay senescence and often result in higher grain yield [12–18].

In barley, the genetic diversity and physiological effects of the two NAM-genes are less known. Within *HvNAM-1*, Distelfeld et al. [10] reported two single nucleotide polymorphisms (SNPs) associated with amino acid differences between the high- and low-protein varieties ‘Karl’ and ‘Lewis’, respectively. The associated deposited sequences (EU368851 and EU368852), however, differ at three positions. Fritsch et al. [19] compared 'Karl' with 'Clipper' and reported three SNPs in *HvNAM-1*. Although no significant association between polymorphism in *HvNAM-1* and GPC could be detected, Cai, et al. (2013) showed that a single SNP within *HvNAM-2* was associated with GPC. In the same study significant correlations between *HvNAM-1, HvNAM-2* and GPC were found among a world-wide set of accessions [6]. *NAC* genes constitute a large, plant-specific family of transcription factors, many of which have been identified as regulators of senescence in monocotyledons [20]. Similarly to wheat, pleiotropic effects of the *HvNAM* genes on micronutrient content, senescence and yield have been suggested in barley [21, 22]. In gene expression studies, Christiansen and Gregersen [23] found both *HvNAM-1* and *HvNAM-2* to be upregulated during senescence.

In the Nordic countries (Denmark, Sweden, Norway, Finland and Iceland) barley has been cultivated since Neolithic times [24]. The region contains some of the northernmost areas in the world where barley can be cultivated and in the far north of the Nordic region, barley has been the only crop that can be grown successfully. The short growing season in the Nordic countries imposes particular selection pressures on cultivated crops, and natural selection for early maturation has been suggested as a force conserving the *NAM-B1* wildtype allele in many Nordic spring wheats [25, 26]. It is not known whether natural selection has affected *HvNAM* genes similarly.

Both animal feed and malt have been important uses of barley in the Nordic countries during the twentieth century [27]. Consequently, barley varieties have primarily been developed based on agronomic and malting quality traits with much less attention paid to food or feed quality [28]. In wheat, selection for yield was probably responsible for the gradual decrease in the frequency of the *NAM-B1* wild-type allele in Nordic varieties released during the twentieth century [26]. Should *HvNAM* genes have a pleiotropic effect similar to that of *NAM-B1* a corresponding shift in genetic diversity can be expected.

In this study we have investigated the genetic diversity of *HvNAM-1* and *HvNAM-2* in Nordic barley, a part of the global germplasm where the *HvNAM* genes have not been studied previously. The aim was to correlate it to differences in protein and micronutrient content as well as grain size. We also investigated whether the expression of the two *HvNAM* genes showed any correlation with protein and micronutrient content or grain size. In addition, we were interested in how nutritional and yield traits changed in Nordic barley varieties released during the course of the twentieth century.

## Results

### Nutritional content and its association with Thousand Grain Weight

Nutritional content was measured in 80 Nordic accessions from four different plant improvement periods and in a high and a low GPC control accession (Table 1). Grain protein content, measured as percentage dry matter, ranged from 6.81% for the accession NGB11311, released in 1990, to 15.25% for the accession NGB9424, released in 1897 (Table 1). Iron (Fe), measured as mg/kg dry matter, ranged from 27.23 for the accession NGB303, released in 1966 to 53.83 for the accession NGB9424. Zinc (Zn), also measured as mg/kg dry matter, ranged from 32.72 for the accession NGB2075, released in 1918, to 49.39 for the accession NGB13022, released in 1994.

**Table 1:** Information about the accessions used in the study. Measurements of nutrient content in grains, thousand grain weight (TGW) and plant height are averages of three replicates

Accession number	Accession name	Country of origin	Row-type	Improvement group (release year)	Protein (% of DM)	Fe (mg/kg DM)	Zn (mg/kg DM)	TGW (g)	Height (cm)
NGB4613	Gammel Dansk	Denmark	2-row	landrace	7.00	46.92	21.08	51.33	73.66
NGB4641	Støvring	Denmark	2-row	landrace	8.19	50.08	23.59	48.00	79.00
NGB6929	Gaffel Dækket	Denmark	2-row	landrace	8.69	46.65	24.10	56.00	99.00
NGB9511	Langeland	Denmark	2-row	landrace	10.62	39.49	24.87	45.56	71.66
NGB2565	Kääs, local Öland	Sweden	2-row	landrace	9.31	47.15	27.62	47.22	74.50
NGB13504	Lantkorn Från Gotland	Sweden	2-row	landrace	10.00	41.67	26.73	42.44	51.00
NGB277	Lähde	Finland	2-row	landrace	8.56	41.53	19.51	44.56	86.33
NGB9529	Lynderupgaard	Denmark	6-row	landrace	7.88	38.40	20.31	39.89	96.83
NGB468	Trysil	Norway	6-row	landrace	7.625	39.08	18.55	43.00	81.00
NGB469	Bjørneby	Norway	6-row	landrace	7.31	40.38	18.89	38.11	82.66
NGB2072	Kr Finset	Norway	6-row	landrace	12.69	51.01	38.30	48.78	64.83
NGB15358	Amble A-Sogne-Fjord	Norway	6-row	landrace	7.56	46.07	20.53	50.67	90.75
NGB6927	Uforaedlet Jämtland	Sweden	6-row	landrace	8.12	38.85	18.76	39.00	93.16
NGB15103	Lulea 31/185	Sweden	6-row	landrace	8.94	47.56	24.96	38.89	79.50
NGB15178	Skaneslet	Sweden	6-row	landrace	10.06	50.32	25.42	38.44	88.66
NGB27	Sarkalahti Me0103	Finland	6-row	landrace	8.00	44.81	23.45	41.22	96.16
NGB308	Veteläinen	Finland	6-row	landrace	8.31	39.97	23.36	42.44	74.16
NGB316	Piita	Finland	6-row	landrace	9.69	48.15	28.00	42.78	91.33
NGB321	Törmälä	Finland	6-row	landrace	9.50	51.48	29.00	42.22	96.00
NGB4619	Opal Abed	Denmark	2-row	cultivar, 1890–1940 (1922)	11.25	47.37	21.68	37.00	82.00
NGB8815	Maja Abed	Denmark	2-row	cultivar, 1890–1940 (1927)	10.19	51.33	38.48	33.44	63.33
NGB9465	Rex Abed	Denmark	2-row	cultivar, 1890–1940 (1913)	8.69	42.60	20.35	48.78	64.33
NGB1480	Gull	Sweden	2-row	cultivar, 1890–1940 (1913)	11.00	37.55	23.14	39.78	93.00
NGB1483	Primus	Sweden	2-row	cultivar, 1890–1940 (1901)	13.50	38.06	25.64	43.33	91.33
NGB9424	Prinsess	Sweden	2-row	cultivar, 1890–1940 (1897)	15.25	48.76	26.01	46.56	86.83
NGB9472	Östgöta Flaettring	Sweden	2-row	cultivar, 1890–1940 (N.A.)	9.50	46.51	18.68	57.78	71.66
NGB8234	Piikkiönohra	Finland	2-row	cultivar, 1890–1940 (1922)	7.56	48.06	25.33	44.00	63.00
NGB9343	Lapinohra	Finland	2-row	cultivar, 1890–1940 (1924)	7.19	48.39	27.01	45.78	93.33
NGB9562	Louhi	Finland	2-row	cultivar, 1890–1940 (1934)	8.56	32.72	16.19	39.00	96.33
NGB4585	Juli Abed	Denmark	6-row	cultivar, 1890–1940 (1909)	8.44	45.61	23.41	45.89	83.83
NGB6273	Karls	Denmark	6-row	cultivar, 1890–1940 (1909)	7.38	33.86	18.04	48.22	73.33
NGB459	Maskin	Norway	6-row	cultivar, 1890–1940 (1918)	9.00	32.68	23.11	53.78	62.00
NGB466	Jotun	Norway	6-row	cultivar, 1890–1940 (1930)	9.62	41.95	27.46	60.56	60.50
NGB2064	Fløya	Norway	6-row	cultivar, 1890–1940 (1939)	8.81	39.37	18.48	51.00	58.50
NGB2075	Mjøs	Norway	6-row	cultivar, 1890–1940 (1918)	7.38	36.55	19.82	33.67	56.16
NGB2077	Polar	Norway	6-row	cultivar, 1890–1940 (1933)	9.81	49.33	23.08	43.89	59.66
NGB6272	Dore	Sweden	6-row	cultivar, 1890–1940 (1932)	8.25	29.05	23.76	56.00	54.66
NGB15238	Vega	Sweden	6-row	cultivar, 1890–1940 (1920)	7.69	N.A	N.A	N.A	N.A
NGB6925	Tammi	Finland	6-row	cultivar, 1890–1940 (1937)	7.62	27.23	27.77	37.56	52.66
NGB13660	Olli	Finland	6-row	cultivar, 1890–1940 (1927)	7.38	41.13	25.40	45.78	73.83
NGB4682	Drost A	Denmark	2-row	cultivar, 1941–1970 (1957)	9.19	44.34	22.69	52.89	62.66
NGB8814	Alfa	Denmark	2-row	cultivar, 1941–1970 (1947)	8.94	46.84	25.32	53.56	59.33
NGB8818	Rigel Abed	Denmark	2-row	cultivar, 1941–1970 (1941)	8.50	43.59	22.03	52.22	63.50
NGB8887	Dana	Denmark	2-row	cultivar, 1941–1970 (1964)	8.88	37.19	28.47	32.00	51.16
NGB9637	Siri	Denmark	2-row	cultivar, 1941–1970 (1969)	11.00	42.68	29.40	52.44	64.83
NGB1493	Domen	Norway	2-row	cultivar, 1941–1970 (1952)	11.69	41.67	29.28	45.22	63.83
NGB2105	Goliat	Norway	2-row	cultivar, 1941–1970 (1947)	11.69	39.13	27.03	46.22	66.16
NGB2106	Møyjar	Norway	2-row	cultivar, 1941–1970 (1969)	7.62	45.50	28.84	45.44	95.00
NGB2663	Särla	Sweden	2-row	cultivar, 1941–1970 (1965)	9.12	N.A	N.A	N.A	N.A
NGB2671	Ingrid	Sweden	2-row	cultivar, 1941–1970 (1956)	8.69	44.27	21.62	38.11	68.50
NGB4611	Balder	Sweden	2-row	cultivar, 1941–1970 (1945)	10.50	49.12	28.14	53.11	71.00
NGB287	Karri	Finland	2-row	cultivar, 1941–1970 (1967)	8.25	51.80	37.60	53.67	69.50
NGB303	Arvo	Finland	2-row	cultivar, 1941–1970 (1966)	10.25	35.74	31.61	22.11	76.16
NGB9554	Helmi	Finland	2-row	cultivar, 1941–1970 (1942)	7.69	53.61	33.12	42.33	65.16
NGB2066	Fræg	Norway	6-row	cultivar, 1941–1970 (1948)	12.25	33.61	25.25	49.78	59.33
NGB2070	Jarle	Norway	6-row	cultivar, 1941–1970 (1960)	11.31	49.54	28.33	47.44	50.16
NGB1487	Åsa	Sweden	6-row	cultivar, 1941–1970 (1949)	8.44	46.39	21.69	43.11	65.16
NGB2658	Fimbul	Sweden	6-row	cultivar, 1941–1970 (1946)	N.A	49.37	25.96	54.78	60.33
NGB291	Otra	Finland	6-row	cultivar, 1941–1970 (1959)	8.25	43.63	28.78	49.56	63.00
NGB328	Pomo	Finland	6-row	cultivar, 1941–1970 (1968)	11.44	34.73	32.96	36.89	48.00
NGB4704	Nordal	Denmark	2-row	cultivar, 1971-present (1971)	7.94	46.93	35.51	34.11	68.66
NGB4718	Caja	Denmark	2-row	cultivar, 1971-present (1979)	8.50	35.48	26.35	42.67	59.66
NGB6305	Alis Abed	Denmark	2-row	cultivar, 1971-present (1985)	7.00	37.69	22.78	37.00	76.66
NGB13068	Hamu	Denmark	2-row	cultivar, 1971-present (1990)	N.A	46.43	24.91	34.89	82.33
NGB16752	Otira	Denmark	2-row	cultivar, 1971-present (1997)	7.25	44.07	20.28	40.67	87.00
NGB1505	Simba	Sweden	2-row	cultivar, 1971-present (1975)	8.94	44.25	21.33	36.56	92.33
NGB1510	Pernilla	Sweden	2-row	cultivar, 1971-present (1979)	8.19	32.07	17.38	39.44	93.00
NGB9944	Ariel	Sweden	2-row	cultivar, 1971-present (1987)	7.88	32.67	21.96	51.67	80.00
NGB12276	Svani	Sweden	2-row	cultivar, 1971-present (1992)	7.19	39.24	24.60	42.22	61.33
NGB13913	Cecilia	Sweden	2-row	cultivar, 1971-present (1998)	7.25	42.92	19.88	56.00	62.17
NGB2084	Yrjar	Norway	6-row	cultivar, 1971-present (1975)	7.56	49.48	22.36	55.22	55.00
NGB6605	Tore	Norway	6-row	cultivar, 1971-present (1986)	8.00	33.63	19.06	48.22	50.33
NGB11311	Arve	Norway	6-row	cultivar, 1971-present (1990)	6.81	30.14	19.63	51.67	57.83
NGB13022	Olsok	Norway	6-row	cultivar, 1971-present (1994)	10.06	40.58	20.88	52.67	46.50
NGB16729	Fager	Norway	6-row	cultivar, 1971-present (2000)	10.19	36.72	19.23	51.67	63.00
NGB296	Suvi	Finland	6-row	cultivar, 1971-present (1973)	10.81	48.74	24.55	46.89	58.33
NGB4011	Arra	Finland	6-row	cultivar, 1971-present (1982)	9.25	46.57	21.52	46.33	46.00
NGB9942	Nord	Finland	6-row	cultivar, 1971-present (1988)	13.00	31.11	23.25	42.78	57.50
NGB10654	Loviisa	Finland	6-row	cultivar, 1971-present (1996)	10.44	49.39	41.66	57.33	62.00
Rolfi	Rolfi	Finland	6-row	cultivar, 1971-present (1997)	8.56	53.61	35.72	54.78	48.33
CIho 15,487	Karl	USA	6-row		6.88	44.18	30.54	46.67	56.16
CIho 15,856	Lewis	USA	2-row		8.38	53.83	37.15	24.78	61.83

The low GPC control accession, CIho15487, had the second lowest protein content among the accessions. In contrast, the high control accession, CIho15856, had a protein content that was lower than the mean protein content of all accessions. The high control accession had a Fe content that was in the highest quartile but the low control accession had a Fe content that was higher than the mean among all accessions. For Zn both control accessions had values that laid between the first and the third quartile.

Thousand Grain Weight (TGW) was negatively correlated with protein and Zn content in two-row accessions (protein c = -0.652, *p* < 0.001; Zn c = -0.570, *p* < 0.001) and it was positively correlated with Fe (c = 0.421, *p* < 0.01) and Zn (c = 0.681, p < 0.001) in six-row accessions. Average plant height was in six-row accessions negatively correlated with both Zn (c = -0.404, *p* < 0.05) and protein (c = -0.366, *p* < 0.05). Remaining nutritional measurements were not significantly correlated with either TGW or average plant height (all *p* > 0.05).

### Agronomic trait and nutritional differences among improvement stages and row-types

**Fig. 1:**
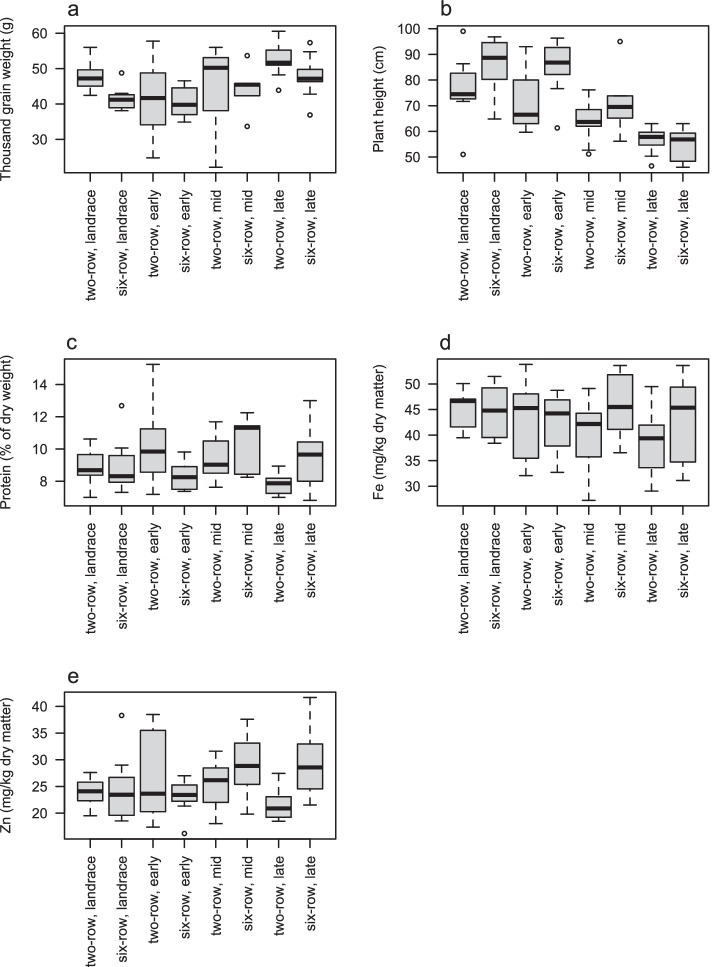
Boxplot of improvement groups and row types for **a** thousand grain weight; **b** plant height; **c** protein; **d** Fe **e** Zn. Improvement groups consist of landraces and cultivars belonging to an old (released 1890–1940), mid (released 1941–1970) and late (released 1971- 2000) group respectively. The boxes display median value and the first and third quartile, the whiskers the maximum and minimum value

We next compared how agronomic traits and nutrient content varied among improvement groups and row-types in Generalized Linear Model (GLM) analyses (Fig. 1). TGW was significantly higher in both landraces (mean 44.92 g, *p* < 0.05) and in late improvement accessions (mean 49.96 g, *p* < 0.001) than in the early improvement accessions (mean 40.38 g), and higher in two-row (mean 46.67 g) than in six-row barley (43.30 g, *p* < 0.05) in a GLM including both improvement group and row type (*glm(TGW* ~ *improvement_group* + *row_type)*) (Fig. 1a).

Plant height was lowest in the accession NGB4011 (46.00 cm) and highest in the accession NGB6929 (99.00 cm). In the corresponding GLM of average plant height, height differed between two-row (mean 66.27) and six-row barley (mean 75.46, *p* < 0.01) and was significantly lower in mid (mean 66.15; *p* < 0,01) and late (mean 55.51; *p* < 0.001) improvement accessions than in early improvement accessions (mean 79,00) (Fig. 1b). Neither protein, Fe nor Zn content differed between improvement stages or row-types in GLM analyses (all *p* > 0.05) (Fig. 1c, d, e).

### Haplotypes and genetic diversity

Sequencing of the complete *HvNAM-2* gene revealed that all the Nordic accessions were genetically identical to the high GPC control accession CIho15856. *HvNAM-1* also had low genetic diversity, but three different haplotypes were detected among the Nordic *HvNAM-1* sequences (Table 2). The majority of the sequenced accessions were identical to the high GPC control accession, CIho15856. In the two additional haplotypes, NGB9343 (an accession from the early improvement group) carried a C at position 1063 and NGB6929 (a landrace accession) carried an A at position 1167 using the coordinate system of GenBank accession DQ869678.1. The former of the two substitutions was synonymous (Pro—> Pro) while the second resulted in a tolerated amino acid substitutions (Gly—> Asp), with a SIFT score of 0.15. While NGB6929 had average mineral and protein content and kernel size, values for NGB9343 fell below the lowest quartile for N, Fe, Zn and kernel size. The three differing nucleotides in the low-GPC control accession CIho15487 were not found in any other accession.

**Table 2:** Consensus sequence and variable positions for three detected haplotypes in *HvNAM-1*

Accession	234	544	1063	1167	1433
consensus	G	G	G	G	G
NGB9343			C		
NGB6929				A	
Clho15487 (low GPC control)	C	C			A

### Gene expression

**Fig. 2:**
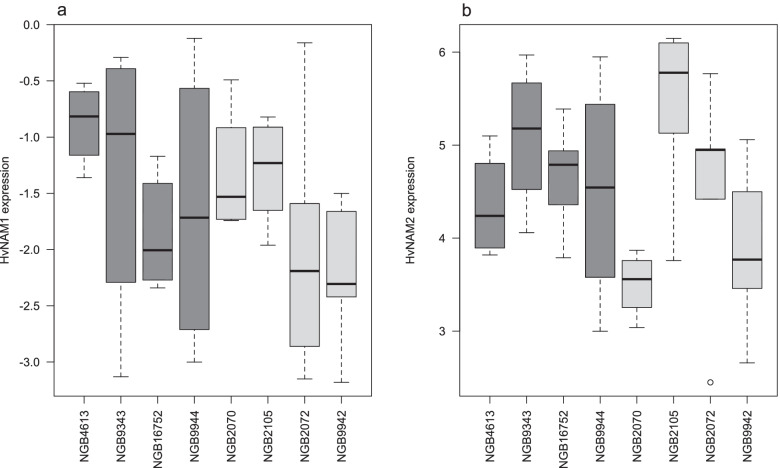
Relative expression of **a** HvNAM-1 and **b** HvNAM-2 respectively in flag leaves probed 22 days after spike emergence. Dark grey boxes denote high protein content accessions and light grey boxes denote low protein content accessions. For details see Supplementary Table 1

Gene expression of *HvNAM-1* and *HvNAM-2* was determined in a subset of eight accessions, representing low and high grain protein content, using qRT-PCR. In the flag leaf, probed 22 days after spike emergence, *HvNAM-1* was expressed significantly more (had lower Cq values) than *HvNAM-2* (paired t-test, *p* < 0.001). There was no significant correlation between the expression levels of the two genes (*p* = 0.16, c = 0.23). Among the analysed accessions, *HvNAM-1* had the highest expression (lowest Cq values) in the low protein content accession NGB9942 and the lowest expression in the high protein content accession NGB4613 (Fig. 2). *HvNAM-2* had the highest expression in the low protein content accession NGB2070 and the lowest in another low protein content accession, NGB2105. The expression levels of neither *HvNAM-1* nor *HvNAM-2* were correlated with either nutrient content (protein, Fe, Zn), or TGW (all *p* > 0.05).

## Discussion

### Nordic barley improvement changes during the twentieth century

Barley was one of the first target species when modern plant improvement was initiated in the Nordic region in the 1890s [27, 29]. Improvement of two- and six-row barley have since been carried out independently of each other, with the former being preferred by the malting industry and for cultivation in the south, and the latter primarily as feed barley and for more northern cultivation areas [27]. In contrast with the six-row barley grown in the north, breeding of malting barley also needed to meet quality demands from the malting industry such as low protein, high extraction capacity and even germination ability [28]. Among the most recent cultivars, GPC is also lower in two-row than in six-row varieties. Of the traits measured in this study, TGW and plant height were the only traits for which the row-types differed among the improvement groups, with larger grain and shorter straw-length for two-row barleys. Neither of the values for nutritional contents showed any significant differences in GLM analysis.

A reduction in plant height has been an improvement target not only in barley but also for other cereals during the twentieth century [30]. Plant height in our set of Nordic barley, although following this general trend, was not consistently reduced when comparing more recent improvement groups with older ones. Bertholdsson and Brantestam [31], however, showed that much of the detected effects of plant improvement occurred late in the history of plant improvement, which is supported here by shorter plant heights in accessions from the late improvement group, in both row type groups. In our set of accessions, really tall varieties (> 90 cm) were primarily released before 1950. Accessions released during the second half of the twentieth century were more uniform in height with ever shorter straw lengths (Fig. 1b).

In wheat, worldwide breeding from the 1960s and onwards drastically reduced straw length and increased harvest index and grain yield [32]. As grain yield is often negatively correlated with GPC in wheat [33–38], the raised harvests came at the cost of lower GPC, as well as lower and micronutrient content [39, 40]. Similar trends for GPC have been reported in Swedish spring wheats [17].

In our set of Nordic barley we could not detect any differences in nutrient content among the improvement groups, or between row-types. As in wheat, harvest index increased dramatically during twentieth century Nordic barley breeding [31]. Although we were unable to obtain estimates of yield, we note high TGW and short straw length in the late improvement group. Our results indicate that high TGW has not been obtained at the cost of lower GPC. We found no consistent negative correlation between nutritional value and TGW in the accessions studied, although TGW was negatively correlated with protein and Fe in two-row barley.

### Links between *HvNAM* genetic diversity and nutrient content

We found low diversity of *HvNAM* in our material, in agreement with Jamar et al. [9], in their screen of European cultivars, but contradicting the Wang et al. [41] study of *HvNAM-1* in European landrace barley. All but two of the Nordic accessions carried the haplotype Hap7 of Wang et al. [41], the second most common haplotype of that study. The lack of diversity in our material reflects the homogeneity of Nordic barley in a European context [42]. The low genetic diversity prevented us from investigating associations between genetic diversity in either *HvNAM* gene and nutrient content. Furthermore, the only accessions where SNPs were observed are unlikely to elucidate the function of *HvNAM-1*. In NGB9343, the SNP did not cause an amino acid substitution and thus, although this accession had low protein, Fe and Zn, we cannot link this to variation in *HvNAM-1*. The SNP in NGB6929 caused a tolerated aminio acid substitution, but had intermediate grain protein and micronutrient content. The substitution hence caused no detectable phenotypic change in these traits.

Failing to find genetic diversity in eleven studied barley accessions Jamar et al. [9] suggested that differences in protein content could be caused by differential expression of in particular *HvNAM-1*. Similar to Distelfeld et al. [21] our analysis of the expression of the *HvNAM* genes, carried out in a subset of our accessions, showed higher expression of *HvNAM-1* than *HvNAM-2*. However, we could not detect any significant association between gene expression and nutrient content.

### Evolution of the *HvNAM* genes

The low genetic diversity of the *HvNAM* genes meant that no loss of genetic diversity during twentieth century breeding, similar to *NAM-B1* in wheat as described by [17] could be seen. The low diversity, in particular in the intronic regions which were invariant, of both *HvNAM* genes tentatively suggest a past selective sweep, similar to the one described for *NAM-B1* in wheat [43]. What the selected trait(s) could have been can only be speculated upon, but if selection was at least moderately strong, the sweep is expected to be quite wide as a consequence of the selfing habit of barley, possibly containing multiple genes. It is not unlikely that regulatory regions of the two genes also show limited genetic diversity and that the differences in expression are expected to be minor. What little diversity was detected occurred in a landrace and an early accession tentatively suggesting more diversity could be present in unimproved Nordic barley. In their more variable set of landrace barley, Wang et al. [41] were, however, unable to detect any statistically significant signals of selection on *HvNAM-1*.

The GPC of 49 of the 80 the accessions studied here was higher than that of the high protein control accession, CIho15856, used in this and other studies of GPC in barley [7, 8, 10]. Only a single accession, NGB11311, had a lower protein content than the low protein control. This is surprising given that barley improvement schemes in Nordic primarily have targeted malting rather than feed quality [28] and that high protein content is not necessarily desired for malting. In wheat it has been suggested that selection for fast maturation time acting on *NAM-B1* has resulted in preservation of the high protein content allele [17, 26]. Earlier senescence in high GPC accessions has similarly been suggested as a cause for negative correlations between GPC and yield in barley [22]. Many barley NAC genes, including *HvNAM-1* are also active in the senescence process [20, 23] and selection for early maturation may have pleiotropically kept protein content high. This, however, seems unlikely to be the full explanation. We note that the Hap7 of Wang et al. [41], the most common one in our Nordic barley, is frequently occurring also in climates where much longer growing seasons are expected than in the Nordic region [41] and where fast maturation is not needed to the same extent.

Although *HvNAM-1* and *HvNAM-2* were almost completely void of genetic diversity, our panel of accessions was not invariant in terms of GPC or micronutrients. It is likely that at least some nutrient variation is caused by genetic effects, although it is clear that the differences detected cannot be attributed to genetic variation in the *HvNAM* genes. Instead, it is likely that evolution in other loci has given rise to these differences. In their GWAS of protein content Cai et al. [6] not only found associations with *HvNAM-1* on chromosome 6H and *HvNAM-2* on chromosome 2H. In addition, multiple markers, in many instances within 10 cM of each other, on chromosomes 1H, 3H, 5H and 7H were also found to be associated with protein content. QTL mapping of protein content in Australian and North American two-row barley has similarly showed the trait to be under polygenic control with QTLs identified mainly on 2H, 4H, 5H and 6H [3]. Future studies should evaluate these regions in Nordic barley and explore the material for associations between markers and protein content, to gain a more complete picture of the evolution of differences in nutrient variation.

## Conclusions

We find reduced straw length and increased harvest index during twentieth century Nordic barley breeding but no corresponding reduction of grain protein or micronutrient (Zn, Fe) content during the same time period. The genes *HvNAM-1* and *HvNAM-2* have very little genetic diversity in the Nordic barley gene pool and their expression was not correlated with grain nutrient content. The material nevertheless does show substantial variation in grain nutrient content that could be exploited for further breeding. Genetic variation of *HvNAM-1* and *HvNAM-2* could also be introduced from other sources, or altered by mutation breeding [44], to contribute additional genetic control of grain protein content.

## Methods

### Plant material

Eighty accessions of barley from the Nordic region, selected from the set investigated by Kolodinska Brantestam et al. [45], were obtained from NordGen (the Nordic Genetic Resource Center) (Table 1). The accessions consisted of two- and six-row barley from four improvement groups: Landraces and cultivars belonging to an old (released 1890–1940), a mid (released 1941–1970) and a late (released 1971- 2000) group respectively. Accessions from all Nordic countries (with the exception of Iceland) were equally represented within each improvement group.

Two barley varieties, CIho15487 (‘Karl’) and CIho15856 (‘Lewis’), received from the Germplasm Research facility of the Small Grains Collection, US were used as controls. CIho15487 has low GPC while CIho15856 has high GPC. CIho15487 and CIho15856 are known to differ in two SNPs (Single Nucleotide Polymorphism) located in *HvNAM-1* [10].

From the set of accessions, four high protein accessions (NGB4613, NGB9343, NGB16752 and NGB9944) and four low protein accessions (NGB2070, NGB2105, NGB2072 and NGB9942) were chosen for expression analyses of *HvNAM-1* and *HvNAM-2*. These eight accessions represented all Nordic countries and three of the four improvement groups. The oldest cultivar group was represented by a single accession.

### Plant cultivation

The cultivation experiment was laid out in a randomized block design with three replicates and conducted in a greenhouse with supplementary lighting to create a daylength of 16 h. Day temperature was set to 22 °C and night temperature to 16 °C. The plants were grown in pots 12 × 12 × 12 cm in size, filled with a fertilized peat compost mixture originally containing ~ 200 mg N per pot and essential macro- and micronutrients (g m^−3^, N 140, P 85, K 150, Fe 4.7, Zn 1.2). Three seeds were sown per pot and reduced to one plant per pot after 17 days. After 17 days the plants were also supplied with additional fertilizer (Algomin Professional Gardening) containing (per pot) 40 mg N, 50 mg P and 110 mg K. The plants were watered with tap water as needed to keep the soil moist throughout the experiment.

Height was measured from the soil surface to the base of the primary spike at full maturation. Grains were harvested manually at full maturity. Three spikes from each plant (less if enough spikes were not available) were harvested. The grains were then dried in a heating cupboard for 48 h at 60 °C and the dry weight was measured. The dried grains were threshed to remove all chaff and counted to obtain 30 grains for estimation of thousand grain weight (TGW). The grain samples were analysed for nitrogen (Leco Corp, Lakeview Avenue, United States), iron and zinc content (Inductively Coupled Plasma atomic emission spectrometry) at Agrilab AB in Uppsala, Sweden. Nitrogen content was converted to protein content through multiplication with 6.25.

The eight accessions used for RNA analysis (NGB4613, NGB2072, NGB9343, NGB2070, NGB2105, NGB16752, NGB9942, NGB9944) were grown as above in single pots, one plant per pot, with six replicates placed in a complete randomized design and surrounded by one row of plants to minimize edge effects. The time points for spike emergence were noted and 22 days later the flag leaves were collected from all replicates, flash frozen in liquid nitrogen and stored at -80 °C until used.

### Genetic analysis

DNA was extracted from the different accessions using the Qiagen DNAeasy Plant Mini Kit (Qiagen, Germany) according to the manufacturer's recommendation. *HvNAM-1* and *HvNAM-2* were amplified in all accessions using PCR. The PCR reaction contained 1 µl of template DNA, 16 µl MilliQ water, 2 µl 10 × DreamTaq Buffer (Thermo Scientific), 0.4 µl 10 mM dNTP (Thermo Scientific), 0.2 µl of 10 µM of each forward and reverse primer and 0.2 µl DreamTaq polymerase (Thermo Scientific). The primers used for amplification of *HvNAM-1* were: HvNAM1F 5’-ATGGGCAGCCCGGACTCATCCTCC-3’ and HvNAM1R 5’-TACAGGGATTCCAGTTCACGCCGGAT-3' and for *HvNAM-2*: HvNAM2F 5’-ATGGGCAGCTCGGACTCATCTTCC-3’ and HvNAM2R 5’-TCAGGGATTCCAGTTCACGCCGGA-3’. These primers, together with internal sequencing primers (HvNAM1SeqF 5’-GCATGAGTACCGCCTCAC-3’ and HvNAM1SeqR 5’-GTGAGGCGGTACTCATGC-3’ for HvNAM-1 and HvNAM2SeqF 5’-GCAGTAACCGATCTCCGTATTT-3’ and HvNAM2SeqR 5’-GGAGATCGGTTACTGCTTGAC-3’ for HvNAM-2, respectively) were used for Sanger sequencing. Sequencing was carried out by Macrogen Europe, the Netherlands.

RNA was isolated from frozen (-80 °C) barley flag leaf tissue using RNeasy Mini Kit as described in [46]. The extracted RNA was diluted to 20 ng/µl and samples with a lower concentration were discarded, leaving four to six biological replicates per accession (Supplementary Table 1). The primers used for qPCR were for *HvNAM-1*: HvNAM-1QF 5'- CGGCAGTATGTCGCTGTCATCC-3' and HvNAM-1QR 5'-ATGGCGTTCACCGCATTGCC-3', for *HvNAM-2*: HvNAM-2QF 5'-CGTATGCCACAGCGTGCATGAC-3' and HvNAM-2QR 5'-CTGGTGATGGAGCAGTGAAGCG-3' and for *SplFact2*: SplFact2QF 5'- GAAGGATGAGTAGGCGCTGG-3' and SplFact2QR 5'-CTGGGAGGTTCCCAACGTAA-3'. QuantiFast SYBR Green RT-PCR (Qiagen AB, Germany) kit was used for the one step qPCR with the following cycling conditions: 10 min at 50 °C for reverse transcription followed by 5 min at 95 °C for Taq activation, and thereafter 40 cycles of 10 s at 95 °C followed by 30 s at 60 °C. To verify for absence of amplifying DNA in the RNA samples negative controls with no reverse transcriptase added in the reaction mixture were run for all samples. Negative controls without template were also run for each primer combination. Amplification of *HvNAM-1*, *HvNAM-2* and the reference gene *SplFact2* were run separately but with two repeated reactions for cross referencing between plates. Negative control qPCRs all resulted in no amplification. Melt curve analyses were normal except for one replicate from the accession NGB4613, which was left out from downstream analyses.

### Data analysis

DNA sequences were aligned and manually corrected using the software Geneious (v 6.0.5). SIFT scores for predicting effects of nonsynonomous sequence variation was calculated using the SIFT web server [47]. R (v 3.5.1) was used to create plots and for performing statistical analyses. Data on thousand grain weight (TGW) and nutrient content could not be obtained for the accessions NGB2658 and NGB13068 and these accessions were hence left out of analyses of TGW and nutrient content. Nutrient measurements carried out on samples weighing less than 30 g were also excluded. Correlations were tested using the *cor.test* command. Generalized Linear Models (GLM, *glm* command) was used to test for differences between row types and improvement groups.

For the qPCR data analysis, the LC480Conversion program (https://www.medischebiologie.nl/files/) was used to convert the raw expression data from the Light Cycler 480 (Roche). The converted data was further analysed with LinRegPCR program to obtain PCR efficiencies and Cq values. Cq values were calibrated across plates and then normalized against the *SplFact2* gene expression using GenEx (MultID Analyses, Sweden).

## Supplementary Information

**Additional file 1. **

## Data Availability

The datasets generated and/or analysed during the current study are available in the GenBank repository, https://www.ncbi.nlm.nih.gov under accession numbers OM416539 - OM416620 (*HvNAM-1*) and OM416621 - OM416702 (*HvNAM-2*).
